# Wettability and Mechanical Properties of Red Mud–Al_2_O_3_ Composites

**DOI:** 10.3390/ma17051095

**Published:** 2024-02-28

**Authors:** Yongliang Chen, Anmin Li, Shiwei Jiang

**Affiliations:** 1 School of Resources, Environment and Materials, Guangxi University, Nanning 530004, China; 2115301009@st.gxu.edu.cn (Y.C.); 2215391037@st.gxu.edu.cn (S.J.); 2 State Key Laboratory of Featured Metal Materials and Life-Cycle Safety for Composite Structures, Guangxi University, Nanning 530004, China

**Keywords:** red mud, composites, mechanical properties, wettability

## Abstract

In 2023, the global production of new red mud is expected to reach nearly 200 million tons, but less than 10% of it is currently being utilized in an environmentally friendly manner. To reduce the sintering temperature of alumina ceramics, a sintering aid method is used, as high-purity alumina ceramics require a solid-phase sintering temperature of over 1700 °C. The metal oxides present in red mud are necessary components for high-performance composite alumina ceramics. Composites were obtained by mixing and sintering red mud and Al_2_O_3_. This study focused on the mechanical properties and wettability of these composites. The results indicated that the 10% red mud–Al_2_O_3_ composite exhibited the highest hardness (20.12 GPa) and flexural strength (346 MPa). This is attributed to the formation of a mineral phase dominated by CaAl_12_O_19_, generated by the red mud during the sintering process, which filled the pores and reduced porosity. The surface energy of the red mud–Al_2_O_3_ composite was the highest at room temperature and high temperature, reaching 49.60 $mJ\cdot m^{-2}$ and 1164.7 $mJ\cdot m^{-2}$, respectively, indicating that it has better stability at both room and high temperatures. This study provides an important fundamental basis for the application of red mud–alumina composites to replace alumina-based composites in the field of construction materials, molten metal filters, ceramic cleavers, etc.

## 1. Introduction

Red mud, which is a solid waste product of the Bayer process that occurs during alumina production, causes serious danger to human health and the environment [1]. Many scientific researchers have found many applications for red mud. Cyril Scribot et al. chose to use modified red mud doped with natural clay to make ceramics, and found a quasi-linear regression between the densification and the amount of modified red mud, and obtained ceramic samples containing 10% modified red mud with a compressive strength of 64.9 MPa by sintering it at 1015 °C [2]. Vsévolod Mymrin et al. utilized three types of industrial wastes, namely red mud, steel slag, and printed circuit boards, to prepare a new ceramic composite with a flexural strength of 15.39 MPa [3]. Dejian Pei et al. used red mud, foliated feldspar, talc, shale, and quartz to sinter ceramics at 1080 °C. They found that Na^+^-solidified calcium feldspar acted as a co-solvent, promoting the formation of the liquid phase and facilitating a lower optimal sintering temperature. Hematite, residual quartz, and generated pyroxene formed skeleton phases in ceramics during the high-temperature sintering stage. In particular, pyroxene helps to increase flexural strength, while slow cooling facilitates the precipitation of more pyroxene and a little calcium feldspar during the sintering process, resulting in SiO_2_-Al_2_O_3_-CaO-MgO ceramics with a flexural strength of 115.88 MPa [4,5]. Wei Wang et al. succeeded in producing low-cost ceramics by adding kaolinite to red mud, where 6% (NH_4_)_6_Mo_7_O_24_ was used as a catalyst to promote the growth of mullite. Fei Peng et al. used SiO_2_ + Na_2_O and SiO_2_ + Na_2_O + B_2_O_3_ as additives. They melted samples and poured them onto an iron plate at room temperature. The mixture was then held at 650 °C for 1 h. This process resulted in the production of two different nanocrystalline glass–ceramics from red mud, each with different additives. Both glasses contained nanocrystalline phases, which were triggered by impurities present in the red mud as a nucleating agent. The main crystalline phase was wollastonite (Ca_3_Si_3_O_9_). This can be explained by a phase diagram of the CaO-Al_2_O_3_-SiO_2_ system. The average crystal size of both microcrystalline glasses is below 100 nm. The crystals are uniformly dispersed in the parent glass. Both microcrystalline glasses exhibit good mechanical properties [6,7]. Ye et al. produced microcrystalline glass using a combination of red mud, waste glass, and dolomite. The optimum foaming temperature was found to be 1130 °C [8]. Microcrystalline glass was prepared by Guo et al. by sintering a mixture of red mud, fly ash, Na_2_B_4_O_7_·10H_2_O, and Na_3_PO_4_ at varying temperatures and holding times [9]. Liu et al. made microcrystalline glass with a homogeneous pore distribution from a mixture of red mud, lead and zinc tailings, and silica sand, and its thermal conductivity, mechanical properties, and chemical stability were greatly affected by the sintering temperature, and the comprehensive properties of the microcrystalline glass obtained by sintering it at 970 °C gave it the potential to be a thermal insulation material [10]. In recent years, glass ceramics, cellular ceramics, and foam ceramics prepared with red mud have also been developed [11,12,13,14,15]. However, the limited application scope of these materials is not enough to use all of the red mud. This issue of the utilization of red mud as a resource remains [16].

Based on studies of Al_2_O_3_ ceramic sintering aids, SiO_2_, CaO, MgO, and some other oxides can form two, three, or more eutectic system with the composition of ceramic materials [17]. Through the addition of sintering aids, the sintering temperature of ceramics can be decreased, and the mechanical properties can be increased. Some research showed some minerals, such as MgAl_2_O_4_, can achieve the same functions [17,18]. Ingrid Ferreira Coutinho et al. used Y_2_O_3_ + CeO_2_-doped Al_2_O_3_. The composites were prepared with a Vickers hardness of 1435 HV and a fracture toughness of 9.7 MPa·m^1/2^ [19]. Tufail Mustafa et al. prepared aluminum oxide nanosheets with a hardness of 19.23 GPa and a strength of 436.43 MPa using graphene oxide as a template using the colloidal method [20]. Al_2_O_3_-SiC-Ni composites were prepared using low-temperature pressureless sintering by D. Firoozbakht et al. The sintering temperature was only 800 °C, the hardness was 56.8 RA, and the strength was 242.5 MPa [21]. The main components of red mud are metal oxides and minerals, which makes it possible for red mud to be used as a sintering aid for alumina ceramics.

In this article, red mud was uniformly mixed with alumina instead of commonly used sintering aids. The effect of red mud content on the mechanical properties of composites was investigated. The mechanical properties of the composites produced were comparable to or better than the same pressureless sintered alumina-based composites, but the fracture toughness was slightly insufficient. The properties were inferior to those of composites produced by hot press sintering and spark plasma sintering. The traditional seated drop method was used to study the surface energy at room temperature and wettability with metal droplets at high temperatures. This work could lead to a wide range of applications for red mud in areas such as refractories, building materials, ceramic cleavers, and molten metal filters, enabling the high-value utilization and large-scale consumption of red mud.

## 2. Materials and Methods

### 2.1. Materials

#### 2.1.1. Raw Materials

The Al_2_O_3_ powder and Cu used in this study were provided by Metallurgical Corporation of China (MCC) New Materials Co., Ltd., Shanghai, China. Glycerol and formamide were provided by Guangdong Guanghua Sci-Tech Co., Ltd., Shantou, China. Table 1 shows the important parameters of the raw materials. Red mud was obtained from Guangxi Huayin Aluminium Co., Ltd., (Baise, China) and separated from Fe_3_O_4_ through strong magnetic separation. The chemical composition of red mud was analyzed by XRF. Table 2 presents the results. The chemical composition of red mud reveals the presence of Al_2_O_3_, Fe_2_O_3_, SiO_2_, and TiO_2_, which are derived from bauxite, while CaO and Na_2_O are introduced through the Bayer process. The red mud was dried in a drying oven at 150 °C for 12 h. The dried red mud pieces were crushed and ground into powder.

#### 2.1.2. Preparation of Red Mud–Al_2_O_3_ Composites

The ceramic powder mixtures for the tile samples were prepared according to the composition provided in Table 3. The red mud composites were produced using the pressureless sintering process. Initially, the raw materials were dried in an electric oven at 150 °C for 2 h. The powder mixtures were then ball-milled at 300 revolutions per minute for 20 h using a high-energy planetary ball mill (QM-3SP4, Nju-Instrument, Jiangsu, China). Following ball grinding, the mixtures were vacuum-dried at 150 °C for 12 h. The composite materials were compressed into a cuboid mold measuring 50 × 15 × 8 mm^3^ using an automatic powder-molding press (MC-30, Changsha MITR Instrument Equipment Co., Ltd., Changsha, China) at a pressure of 15 MPa. Many of the alumina matrix composites were sintered at about 1500 °C for about 2 h. The resulting green bodies were then sintered at 1500 °C for 2 h in a MoSi_2_ electric furnace (KSL-1700-2A, KJ-Group HF-Kejing Anhui, Hefei, China).

### 2.2. Methods

X-ray diffraction (D8 ADVANCE, Bruker, Berlin, Germany) was used for phase analysis of the composites at 40 kV/30 mA. Scanning electron microscopy (S3400, Hitachi, Japan) and an energy-dispersive spectrometer were used to observe the microstructure and morphology of the samples. Relative densities were determined using Equation (1),

(1) $$\rho_{RD}=\frac{\rho_{AD}}{\rho_{TD}}\cdot 100\%$$

where $\rho_{RD}$ is the relative density, $\rho_{AD}$ is the actual density, and $\rho_{TD}$ is the theoretical density.

**Figure 1:**
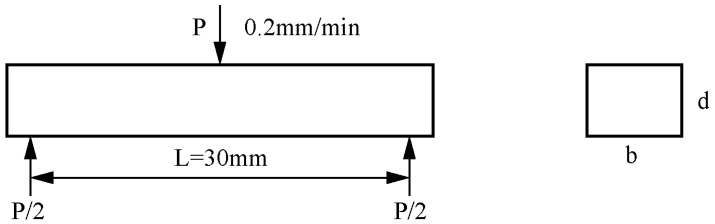
Flexural strength test schematic.

The actual densities were measured using the Archimedes drainage method. The theoretical density was calculated using the law of mixing based on the mass fraction of each component in the composite. Considering that red mud itself is a complex mixture, its density was measured as 2.7 g/cm^3^ using the Archimedes drainage method after the red mud was pressed into blocks. Red mud is a complex mixture, so its true theoretical density is unknown. Here, the theoretical density was measured using compacted red mud blocks. Therefore, the relative density obtained was suitable for comparison with the samples within this study only. The hardness of the sample was measured using a Vickers hardness tester (HV-30, Shanghai, China) with a 10 N load. According to GB/T 4741-1999 [22], the test samples were cut into specimens of approximately 40 × 10 × 5 mm by a ceramic cutting machine. Each specimen was measured by conducting a three-point bending test using a Jilin Guanteng WDW-20 universal mechanical properties testing machine according to GB/T 4741-1999. The specimen size was 40 × 10 × 5 mm, with 8~10 specimens in each group, and the loading speed was 0.2 mm/min during the test. The flexural strength of the sample to be tested was obtained according to Figure 1. The fracture toughness of a material was calculated using the Single-Edge Notched Beam (SENB) method. This involves creating a crack at the midpoint of the specimen and testing it using a three-point flexural fracture test.

**Figure 2:**
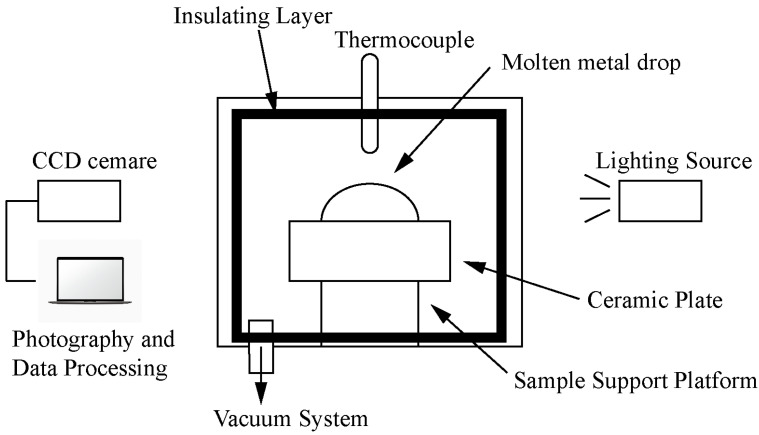
Schematic diagram of wettability equipment.

Figure 2 shows the wettability equipment, which comprises a vacuum tube furnace (KJ-GSL-1800X), a sample stage system, a filming system, and a data processing system. The vacuum tubular furnace’s heating element is a silicon molybdenum rod, and the furnace chamber material is polycrystalline mullite fiber. Before measuring the wettability, the ceramic substrates were polished with 200#, 400#, 800#, and 1000# sandpaper, respectively, and then, the polishing process was completed with diamond polishing paste. The polished ceramic substrates were cleaned in anhydrous ethanol using an ultrasonic cleaner in which surface stains were removed and cleaned.

According to Griffith’s strength theory (σ∝γ), the higher the surface energy, the higher the strength under the same conditions. This can provide a different perspective on the mechanical properties of red mud–alumina composites. The room temperature wettability test was performed using the seated drop method. At room temperature, liquid from a dropper was dropped on the ceramic substrate, which was put into the wettability test equipment, and a CCD camera was used to capture an interface image. The captured image was further processed using ImageJ [23] to obtain the wetting angle and surface energy.

Alumina matrix composites have applications in ceramic cleavers, molten metal filters, etc., so it is necessary to examine their wettability with molten metal. In the field of semiconductor packaging, Cu, as an excellent conductor, is often used as a medium for lead bonding. Therefore, Cu droplets were used as a liquid phase in the high temperature experiments. The specimen preparation for the determination of the wettability of composite ceramics at elevated temperatures was consistent with that at room temperature. The wetting test was performed using the seated drop method, in which the metal was ground and polished into a square with a volume of about 3 × 3 × 3 mm^3^ to remove any possible oxide layer on the metal surface and to ensure that the droplets were symmetrical in shape during the melting process. The metal surface was cleaned with anhydrous ethanol to remove stains before the metal was put into a high-temperature wetting test furnace. A vacuum environment was used for the whole experiment.

## 3. Results

### 3.1. Microstructural and Phase Analysis

**Figure 3:**
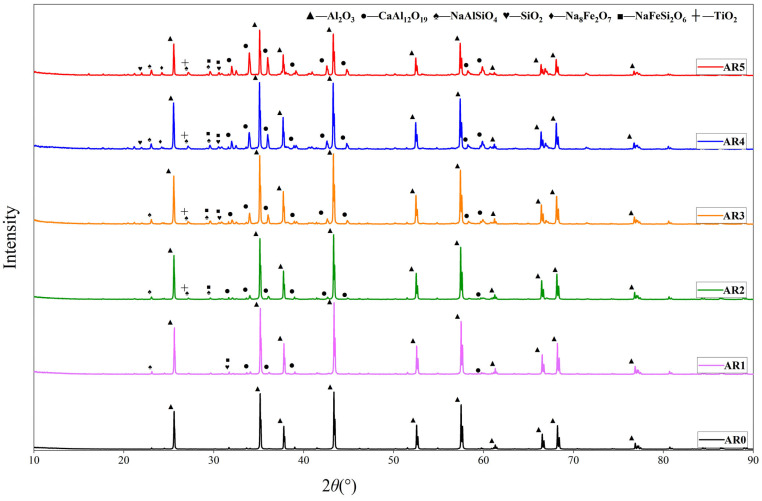
XRD analysis of samples.

From Figure 3, it can be seen that the CaAl_12_O_19_ phase appeared at 34.15° and 36.22° and the intensity of the peaks became larger from AR1 to AR5. The content of CaAl_12_O_19_ in the sintered composites increased with the increase in red mud. In AR0, the peak of CaAl_12_O_19_ did not appear when only alumina was present in the raw material. This suggests that the appearance of CaAl_12_O_19_ is due to the addition of red mud formed after sintering. Because the red mud becomes liquid at 1500 °C, the complex chemical reactions occurring at this temperature can be considered reactions between metal oxides. According to the ternary phase diagram of the Al_2_O_3_-CaO-SiO_2_ system, CaAl_12_O_19_ is produced at a sintering temperature of 1500 °C for the proportion of red mud applied in this experiment. This is in accordance with the XRD experiments. The elemental sodium is present in the composites, mainly in the form of nepheline, sodium pyroxene, and sodium pyroxene. The element silicon is present mainly in the form of quartz, and to some extent, of nepheline and sodium pyroxene. The element titanium exists mainly in the form of rutile. The characteristic peaks are not obvious in AR1 and AR2. According to the XRD results of AR3 to AR5, their characteristic peaks appear gradually. When the red mud content reaches 20% or 25%, the intensity of the characteristic peak changes little.

### 3.2. Mechanical Properties

**Figure 4:**
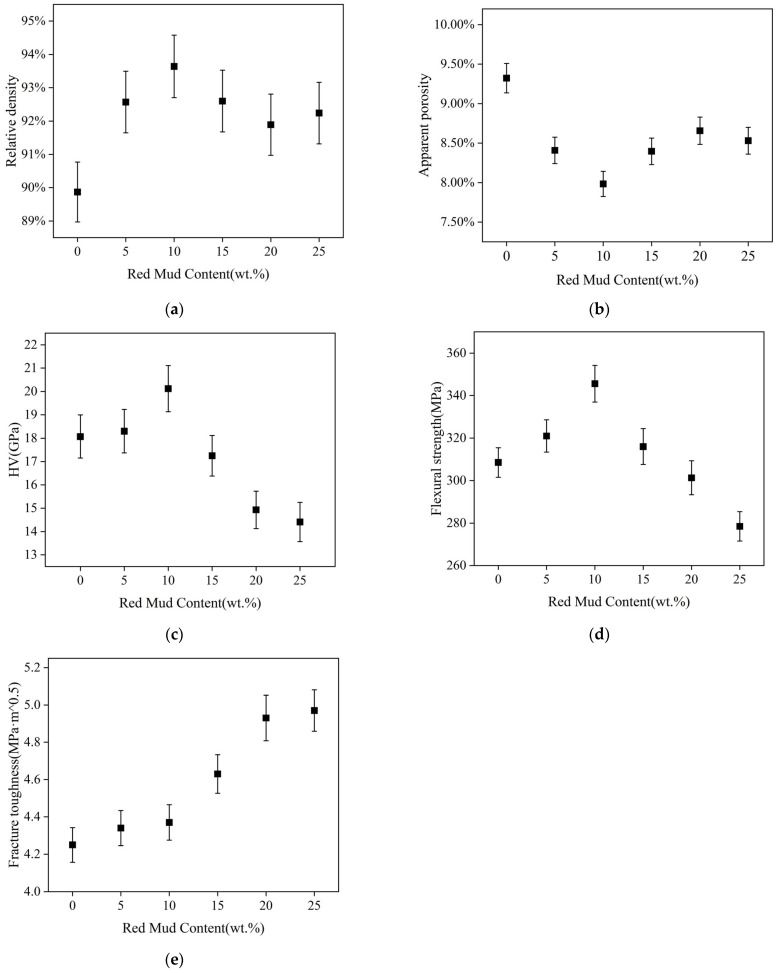
Mechanical properties of samples: (**a**) relative density, (**b**) apparent porosity, (**c**) hardness, (**d**) flexural strength, (**e**) fracture toughness.

Figure 4 shows the relative density, apparent porosity, Vickers hardness, and flexural strength of each sample. The relative density of the sample without red mud is 89.87% ± 0.90%, and the apparent porosity is 9.32% ± 0.19%. The relative density of the red mud–alumina composites increases obviously, and the porosity decreases. This is because at 1500 °C, the appropriate sintering temperature for pure alumina is not reached (about 1700 °C). This shows that the temperature of red mud significantly reduces the sintering temperature of the composite material, so that the ideal sample can be obtained at 1500 °C. A small amount of CaAl_12_O_19_ inhibits abnormal particle growth and reduces pore size between oxidized particles. However, the excessive addition of red mud reduces the relative density of the composite due to volume expansion from in situ CaAl_12_O_19_ formation. Similarly, the porosity of composite materials follows this rule, with the lowest apparent porosity of AR2 occurring with 10% red mud. Excessive CaAl_12_O_19_ production increases the apparent porosity of the composite. Among the composites, AR2 has the best relative density and apparent porosity, which are 93.64% ± 0.94% and 7.98% ± 0.16%, respectively. This shows that compared with other samples, the optimal sintering temperature of 10% red mud composite is closest to 1500 °C.

Calcium hexaluminate (CaAl_12_O_19_) is frequently used as a reinforcing agent to enhance the mechanical properties of ceramic matrix composites. CaAl_12_O_19_ has a stable hexagonal crystal structure, and its microstructure exhibits a regular hexagonal plate-like morphology.

The sample AR2 exhibits the highest hardness value (20.12 GPa) due to its high relative density and low apparent porosity. The number of pores in a ceramic is inversely proportional to its chemical bonds and mechanical properties. As the amount of CaAl_12_O_19_ increases, the hardness values of the ceramics decrease. The inclusion of large amounts of CaAl_12_O_19_ in ceramics leads to an increase in the number of pores [24]. Additionally, CaAl_12_O_19_ is softer than Al_2_O_3_.

Flexural strength shows a similar tendency to hardness. When the red mud content is less than 10%, the flexural strength of the ceramics increases with increasing red mud. AR2 has the highest flexural strength (346 MPa). The continued increase in red mud results in an increase in CaAl_12_O_19_ content. Due to the difference in mechanical properties between CaAl_12_O_19_ and Al_2_O_3_, there are more microcracks inside the ceramics and more pores exist. So, the flexural strength values of AR3 to AR5 are reduced.

With the increase in red mud, the fracture toughness of the composite is increased. The composites exhibit a plate-like morphology of the CaAl_12_O_19_ phase. The toughness of the plate-like composites decreases at small defect sizes and increases at large defect sizes. The incorporation of hexagonal plate-like CaAl_12_O_19_ enhances the toughness compared to single-phase alumina with a similar grain size [25]. Although AR3–AR5 exhibit superior fracture toughness, their flexural strength and hardness are significantly lower compared to AR2. Specifically, while the fracture toughness of AR3 increases by 5.99%, the flexural strength and hardness decrease by 8.55% and 14.26%, respectively. Therefore, overall, AR2 exhibits the best mechanical properties.

The AR2 samples in this experiment had similar or even better mechanical properties compared to the undoped red mud alumina matrix composites. The 5000 wt% ppm ZrO_2_-Al_2_O_3_ composite prepared by Biswajit Baruah et al. had a hardness of 15.89 GPa and a strength of 162.2 MPa [25]; the CaAl_12_O_19_-reinforced Al_2_O_3_-Cr_2_O_3_ composites by Cui. K et al. had a Vickers hardness of 17.02 GPa and a strength of 321 MPa, which are lower than the mechanical properties of AR2 in this experiment [24]. This provides a fundamental basis for the use of red mud-doped alumina composites as a replacement for Al_2_O_3_-based composites used in construction and refractory materials.

**Figure 5:**
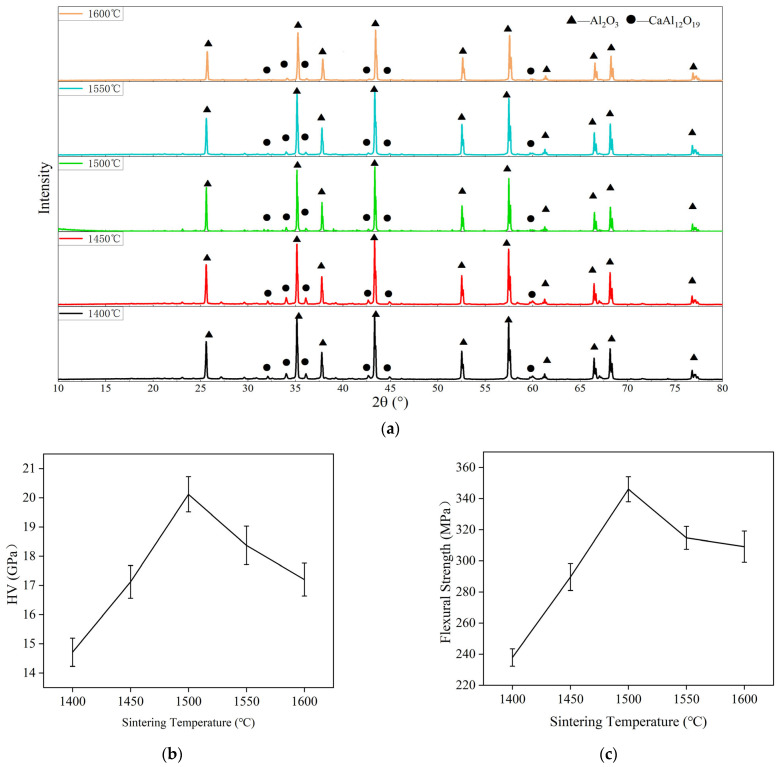
XRD analysis of AR2 samples: (**a**) mechanical properties of AR2 samples: (**b**) hardness, (**c**) flexural strength.

It should be noted that at 1500 °C AR2 has good values for hardness and strength. The hardness and strength of AR2 sintered at different temperatures for 2 h were also measured. There is no change in phase composition. The results (Figure 5) show that the optimum sintering temperature is around 1500 °C. Above 1500 °C, there is a slight decrease in performance. At a sintering temperature of 1600 °C, a large number of cracks appear on the surface of the material, indicating that it overheats at this temperature. Further experiments are required to establish a more accurate optimum sintering temperature and time.

**Figure 6:**
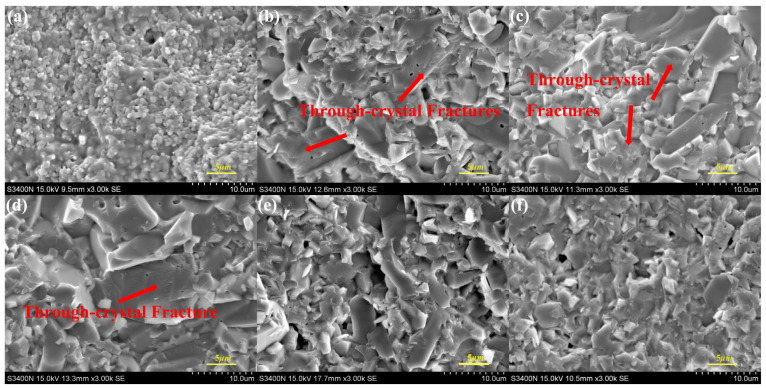
SEM images of fracture morphology of samples: (**a**) AR0; (**b**) AR1; (**c**) AR2; (**d**) AR3; (**e**) AR4; (**f**) AR5.

**Figure 7:**
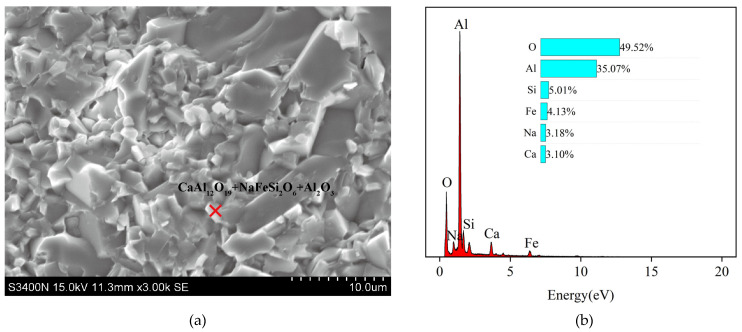
SEM images (**a**) and EDS pattern (**b**) of AR2.

Figure 6 and Figure 7 show the fracture morphology of the specimen. AR0 shows a complete unfired morphology at 1500 °C. This also indicates that the addition of red mud can effectively reduce the sintering temperature of the composites. The fracture cracks of specimens AR1 to AR5 are clearly visible. Cleavage fractures occur in specimens AR1 to AR3, and the cleavage cracks are clearly visible, showing a hairline shape. The material experiences both through-crystal and along-crystal fractures, resulting in a hybrid fracture mode that has a positive effect on the ceramic materials [24,26,27,28,29]. Figure 6 and Figure 7 show that lamellar CaAl_12_O_19_ crystals are embedded in the Al_2_O_3_, resulting in high flexural strength of the ceramics. The hexagonal CaAl_12_O_19_ in AR4 and AR5 gradually thickens, leading to the formation of massive flakes of CaAl_12_O_19_, and an intergranular fracture dominates the fracture mode. The excessive formation of CaAl_12_O_19_ significantly reduces the flexural strength of the ceramics [24,29].

### 3.3. Wettability at Room Temperature

Table 4 shows the wetting angles of the composites’ surfaces with three liquids.

The surface energy can be calculated using the three-liquid tensiometric method proposed by Good and Oss [30]. This theory calculates the surface energy of a polar system using the Young–Good–Girifalco–Fowkes equation [31]:

(2) $$\gamma_{L}\cdot (1+cos\theta )=2(\sqrt{\gamma_{S}^{LW}\cdot \gamma_{L}^{LW}}+\sqrt{\gamma_{S}^{+}\cdot \gamma_{L}^{-}}+\sqrt{\gamma_{S}^{-}\cdot \gamma_{L}^{+}})$$

where $\gamma_{i}^{LW}$ is the Lifshitz–van der Waals interaction; $\gamma_{i}^{-}$ is the surface parameter component of the electron acceptor; and $\gamma_{i}^{+}$ is the surface parameter component of the electron donor.

This study measured the wetting angle and interfacial energy of composites using three known liquids: H_2_O, glycerol, and formamide [30,31]. The solid surface energy was calculated by combining the data in Table 5 with Formula (1). Mathematical instability needs to be considered in the calculation. If the number of conditions of the matrix of the components of the liquid is too high, the matrix is ill conditioned. An ill-conditioned matrix can cause the result to deviate too far from the true values.

The matrix of each component of the liquid has a condition number of 18.66. Considering the large number of conditions, the singular value decomposition (SVD) method is adopted to solve the equation.

From Table 6, it can be seen that from AR0 to AR5, the wetting angle of the red mud–Al_2_O_3_ composite material first increases, and then, decreases. The reason is that the addition of red mud makes the glassy phase appear at the grain boundaries and fills the pores. The interfacial bonding energy between red mud and alumina ceramics is enhanced. The formation of a large interaction within the composite results in an increase in the surface energy of the composite. However, the content of mineral phase in the composites increases with a further increase in the content of red mud. Because of the difference in mechanical properties between the mineral phase and the matrix ceramic, microcracks appear in the ceramic. The interfacial bonding energy between red mud and alumina ceramics is reduced and the surface energy is lowered. From AR0 to AR5, the electron-acceptor component increases, while the electron-donor component decreases. Additionally, the dispersion component increases, and then, decreases. The dispersion component shows an increasing trend followed by a decreasing trend. The trend of the surface energy is positively correlated with the trend of the dispersion component (correlation coefficient *r* = 0.994). This indicates that the material’s surface tension is primarily determined by the Lifshitz–van der Waals component. Al_2_O_3_ has both electron-acceptor and electron-donor components due to its amphiphilic nature. In an alkaline environment, such as that of red mud, alumina behaves as an acid and produces a significant number of electron acceptors. Increasing the amount of red mud leads to greater production of electron acceptors. As alumina is consistently the majority component in the composites tested in this experiment, the electron-acceptor components have a greater impact than the electron-donor components.

### 3.4. Wettability at High Temperature

Table 7 shows the wettability of copper with samples at different temperatures. The contact angles are all greater than 90°, indicating that copper droplets do not wet on red mud–Al_2_O_3_ composite surfaces.

**Figure 8:**
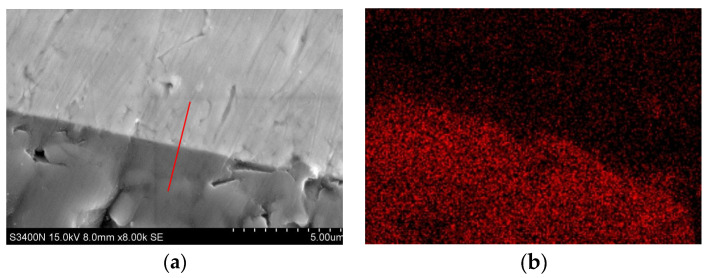

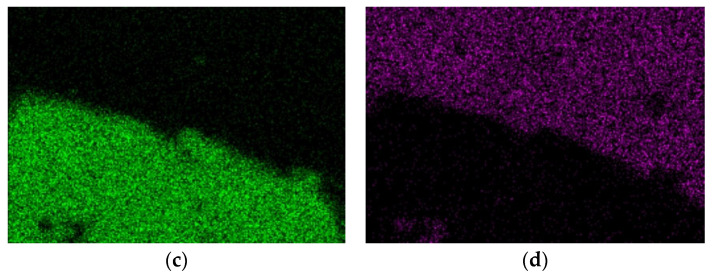
SEM and EDS images of AR2-Cu interface: (**a**) image of interface; (**b**) distribution image of O, (**c**) Al, and (**d**) Cu.

**Figure 9:**
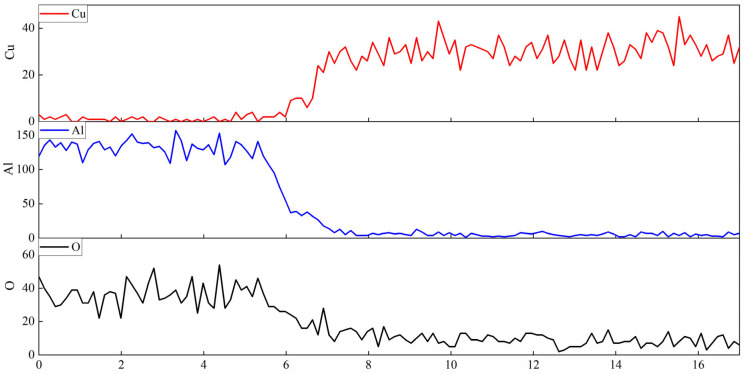
Elemental (O, Al, and Cu) distribution image of AR2-Cu interface.

A cutting machine was used to cut the sample of the copper and composite material after high-temperature wetting in the middle. The Cu-AR2 interface was observed by SEM. As can be seen from Figure 8, the contact surface between the sample and the copper liquid at high temperature is flat, which does not conform to the geometry of the three-phase line for dissolution-driven wetting. According to the results of EDS line scanning (Figure 9), Al and Cu do not appear on the other side, indicating that there is no chemical reaction between the composite material and Cu at high temperature. Only trace amounts of O are diffused into Cu through thermal motion. Therefore, it can be analyzed that the wetting of Cu and the red mud–Al_2_O_3_ composite material at high temperature does not occur, and the liquid component is not adsorbed by the dissolved solution.

**Figure 10:**
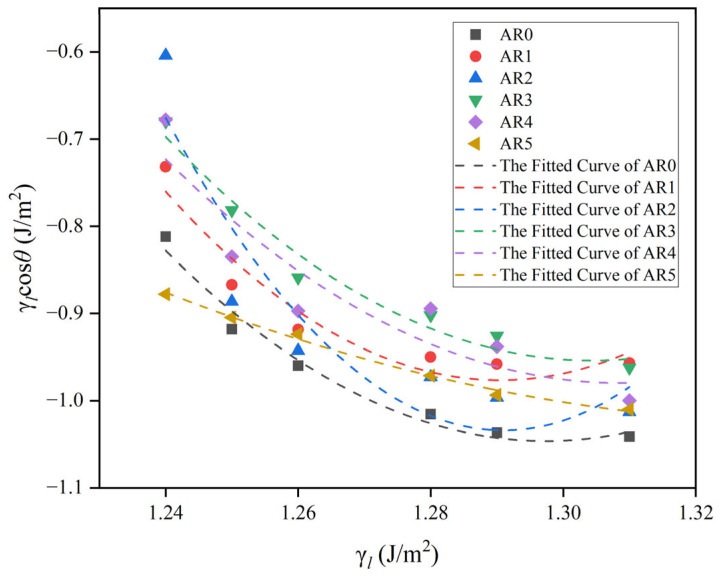
A Zisman plot for the wetting of the red mudAl_2_O_3_ composites by Cu.

The calculation of the surface energy of copper liquids at high temperatures was based on data from K.C. Mill and Y.C. Su [32]: $\gamma_{l}\left(J\cdot m^{-2}\right)=1.32-2.8\times 10^{-4}(T-1358K)$. The surface tension of copper at different temperatures ($\gamma_{l}$) and the values of surface tensions multiplied by the cosine of the contact angles ($\gamma_{l}cos\theta$) are plotted as a Zisman diagram (Figure 10). We used $a\gamma_{l}^{2}+b\gamma_{l}+c$ to fit $\gamma_{l}cos\theta$ [33,34]. The fitting results are shown in Figure 10 and Table 8. According to the theory of the equation of state method for calculating surface tension, $\gamma_{s}=\underset{\theta \to 0}{\lim}\gamma_{l}=\gamma_{l}^{*}$ [34]. The second trinomial obtained from the fit is associated with $\gamma_{l}cos\theta$ = $\gamma_{l}$ to obtain $\gamma_{l}^{*}$ [34]. The $\gamma_{s}$ at 1385 K is obtained. Mathematically, there are two solutions. The smaller one is chosen considering that surface tension is proportional to temperature.

**Figure 11:**
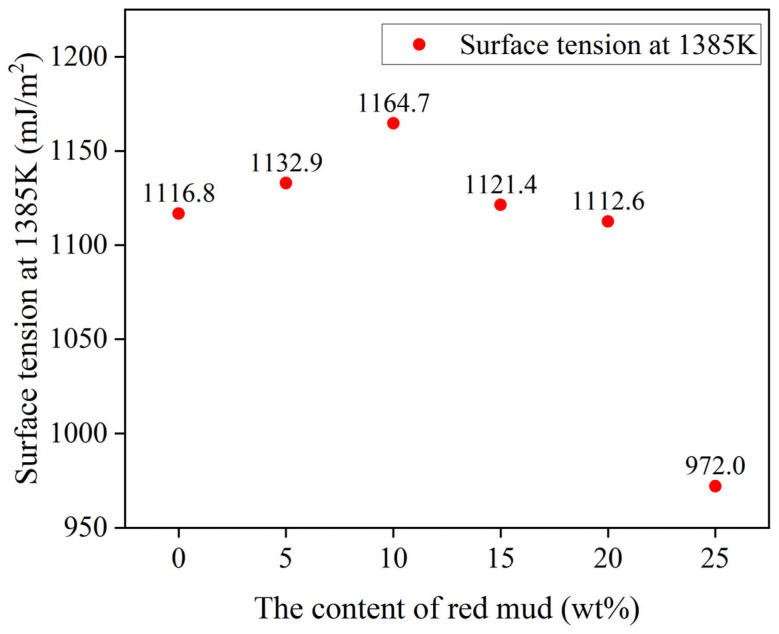
Surface tension of the red mud–Al_2_O_3_ composites at 1385 K.

Figure 11 shows that the surface energy of AR2 is higher than that of the other samples at high temperature. This indicates that AR2 has stronger internal bonding and greater stability at high temperature. In combination with Table 7, AR2 does not wet with Cu at high temperatures, suggesting its potential for use in ceramic cleavers and molten metal filters.

## 4. Conclusions

This study focuses on the mechanical and wettability properties of red mud–Al_2_O_3_ composites. The conclusions are summarized as follows.

(1) The red mud–alumina composites are mainly composed of alumina, CaAl_12_O_19_, and other mineral phases after sintering. The incorporation of red mud reduces the sintering temperature of alumina matrix composites.
(2) The CaAl_12_O_19_ produced by red mud sintering is flaky, which plays a positive role in the mechanical properties of the composite. When the content of red mud is 10 wt%, the composites have the best hardness and flexural strength, but the fracture toughness is low. A further increase in red mud produces a large number of CaAl_12_O_19_, and the mineral phase will significantly reduce the hardness and flexural strength of the composite, but the fracture toughness will have some improvement. These properties make it possible for composites to be used in structural ceramics, refractories, and building materials.
(3) The surface energy of the composites at room temperature reaches its maximum at 10 wt% of red mud. This also indicates that 10 wt% of red mud has the best bonding with the alumina matrix, which is in line with the law of Griffith’s strength theory.
(4) There is a general tendency toward the improvement of properties at high temperatures compared to room temperature. The composites are not wetted with copper liquid. The composite with 10 wt% red mud still has the highest surface energy. Its wettability gives it a base for applications such as ceramic cleavers, molten metal filters, etc.

It is necessary to acknowledge the limitations of this work. The composition of red mud depends on the alumina production process in each region. The plan for future work is to further investigate the sintering temperature and time of the composites to find the best preparation process for the composites, and to test more properties of the composites to investigate wider application scenarios.

## Figures and Tables

**Table 1:** The purity of raw materials.

Raw Materials	Purity
Al_2_O_3_	≥99.9%
Cu	≥99.9%
Glycerol	≥99.0%
Formamide	≥99.5%

**Table 2:** The chemical composition of red mud (wt.%).

Fe_2_O_3_	Al_2_O_3_	SiO_2_	CaO	Na_2_O	TiO_2_	Loss
29.60	18.90	16.90	15.60	11.10	5.78	2.12

**Table 3:** Chemical compositions (wt.%) of the samples.

Sample Code	Al_2_O_3_	Red Mud
AR0	100	0
AR1	95	5
AR2	90	10
AR3	85	15
AR4	80	20
AR5	75	25

**Table 4:** Wetting angle (°) of composites’ surfaces with three liquids.

Sample Code	H_2_O	Glycerol	Formamide
AR0	78.12	55.65	44.21
AR1	74.25	62.42	51.47
AR2	60.98	54.16	40.33
AR3	61.15	61.20	50.86
AR4	53.39	59.50	49.92
AR5	55.69	68.56	62.43

**Table 5:** Surface energy $\left(mJ\cdot m^{-2}\right)$ of liquids with their non-polar, electron-acceptor, and electron-donor components at room temperature.

Liquids	$\gamma_{L}$	$\gamma_{S}^{LW}$	$\gamma_{L}^{+}$	$\gamma_{L}^{-}$
H_2_O	72.8	21.8	25.5	25.5
Glycerol	64	34	3.92	57.4
Formamide	58.0	39.0	2.28	39.6

**Table 6:** Surface energy $\left(mJ\cdot m^{-2}\right)$ of red mud–Al_2_O_3_ composites with their dispersion, electron-acceptor, and electron-donor components.

Sample Code	$\gamma_{L}$	$\gamma_{S}^{LW}$	$\gamma_{L}^{+}$	$\gamma_{L}^{-}$
AR0	41.23	36.36	2.43	2.43
AR1	41.62	37.49	7.59	0.56
AR2	49.60	46.24	16.00	0.18
AR3	40.43	37.45	22.21	0.10
AR4	38.96	35.89	32.46	0.07
AR5	28.18	25.00	40.90	0.06

**Table 7:** Wetting angle (°) of composites’ surfaces with Cu.

Temperature T/(K)	AR0	AR1	AR2	AR3	AR4	AR5
1406	142.9	137.2	140.9	137.5	140.0	140.7
1456	143.4	137.9	140.5	135.8	136.6	140.3
1506	142.7	138.1	139.6	135.0	134.5	139.5
1556	139.5	136.7	138.3	132.9	135.3	137.0
1606	137.3	134.0	135.2	128.8	132.0	136.4
1656	131.1	126.4	119.3	123.4	123.3	135.3

**Table 8:** Values of fitting parameters and corresponding calculation.

Fitting Parameters	AR0	AR1	AR2	AR3	AR4	AR5
a	66.36	84.92	137.97	61.99	54.47	14.63
b	−172.18	−219.19	−356.24	−161.70	−142.57	−39.25
c	110.64	140.46	228.92	104.50	92.31	25.31
*R* ^2^	0.9686	0.8593	0.9947	0.9769	0.9249	0.8454

## Data Availability

Data are contained within the article.
